# Do musculoskeletal ultrasound and magnetic resonance imaging identify synovitis and tenosynovitis at the same joints and tendons? A comparative study in early inflammatory arthritis and clinically suspect arthralgia

**DOI:** 10.1186/s13075-019-1824-z

**Published:** 2019-02-14

**Authors:** Sarah Ohrndorf, Aleid C. Boer, Debbie M. Boeters, Robin M. ten Brinck, Gerd-R. Burmester, Marion C. Kortekaas, Annette H. M. van der Helm-van Mil

**Affiliations:** 1 Department of Rheumatology and Clinical Immunology, Charité-Universitätsmedizin Berlin, Charitéplatz 1, 10117 Berlin, Germany; 2 Department of Rheumatology, Leiden University Medical Centre, Leiden, The Netherlands; 3 Department of Rheumatology, Erasmus Medical Centre, Rotterdam, The Netherlands

**Keywords:** Arthralgia, Early arthritis, Synovitis, Tenosynovitis, Musculoskeletal ultrasound, Magnetic resonance imaging, Rheumatoid arthritis

## Abstract

**Objective:**

Ultrasound (US) and magnetic resonance imaging (MRI) are recommended in the diagnostic process of rheumatoid arthritis. Research on its comparability in early disease phases is scarce. Therefore, we compared synovitis and tenosynovitis detected by US and MRI on joint/tendon level.

**Methods:**

Eight hundred forty joints and 700 tendons of 70 consecutive patients, presenting with inflammatory arthritis or clinically suspect arthralgia, underwent US and MRI of MCP (2–5), wrist and MTP (1–5) joints at the same day. Greyscale (GS) and power Doppler (PD) synovitis were scored according to the modified Szkudlarek method (combining synovial effusion and hypertrophy) and the recently published EULAR-OMERACT method (synovial hypertrophy regardless of the presence of effusion) on static images. US-detected tenosynovitis was scored according to the OMERACT. MRI scans were scored according to the RAMRIS. Test characteristics were calculated on joint/tendon level with MRI as reference. Cut-off for US scores were ≥ 1 and ≥ 2 and for MRI ≥ 1.

**Results:**

Compared to MRI, GS synovitis according to EULAR-OMERACT (cut-off ≥ 1) had a sensitivity ranging from 29 to 75% for the different joint locations; specificity ranged from 80 to 98%. For the modified Szkudlarek method, the sensitivity was 68–91% and specificity 52–71%. PD synovitis had a sensitivity of 30–54% and specificity 97–99% compared to MRI. The sensitivity to detect GS tenosynovitis was 50–78% and the specificity 80–94%. For PD tenosynovitis, the sensitivity was 19–58% and specificity 98–100%.

**Conclusion:**

Current data showed that US is less sensitive than MRI in the early detection of synovitis and tenosynovitis, but resulted in only few non-specific findings. The higher sensitivity of MRI is at the expense of less accessibility and higher costs.

**Electronic supplementary material:**

The online version of this article (10.1186/s13075-019-1824-z) contains supplementary material, which is available to authorized users.

## Background

The value of sensitive imaging methods such as musculoskeletal ultrasound (US) and magnetic resonance imaging (MRI) for disease monitoring in rheumatoid arthritis (RA) is currently being discussed [1]. The diagnostic value of US and MRI in very early disease phases of RA is also being investigated, and there appears to be an agreement on the notion that these modalities have an added value in the diagnostic process [1]. The EULAR imaging taskforce also recommended the use of US and MRI for this purpose without distinguishing between both modalities [2]. These modalities have advantages and disadvantages. MRI is generally considered as the most valid method, yielding reproducible results in a three-dimensional view, and it has the advantage that it depicts bone marrow oedema. Its use is limited by insufficient availability in several centres and higher costs. A disadvantage of US is the machine and operator dependency. Currently available data obtained in patients at risk for RA revealed that US-detected synovitis or tenosynovitis scores (greyscale (GS) or power Doppler (PD)) and MRI-detected synovitis or tenosynovitis scores were predictive for RA development [3–10]. These studies generally used only one modality and did not directly compare findings of both modalities.

Presently, there is limited knowledge whether US and MRI identify the same lesions in the earliest phase of RA. One study compared MRI and US on joint/tendon level in patients with early classified RA; data suggested that MRI is more sensitive than US [11]. The existing studies in early arthritis or arthralgia that performed both MRI and US did not make comparisons on joint or tendon level, did not include the feet, or used low-field MRI [12–15]. In addition, only few studies included tenosynovitis [11–13], and none of them used standardised scoring methods such as the recently published EULAR-OMERACT method for US scoring [16].

Therefore, we aimed to evaluate to what extent both modalities can be used interchangeably in patients at risk for RA. We conducted a cross-sectional study in patients presenting with early inflammatory arthritis (IA) or clinically suspect arthralgia (CSA) and investigated on joint and tendon levels whether US and MRI detected the same inflammatory lesions (synovitis and tenosynovitis).

## Methods

### Patients

Patients that newly presented with early IA or CSA between May and October 2017 at the Leiden rheumatology outpatient clinic were studied. They were consecutively included in either the Early Arthritis Clinic (EAC) cohort or the CSA cohort. Requirements for inclusion in both cohorts are described in reference and supplementary [8, 17].

Both cohort studies were approved by the local Medical Ethical Committee. All patients provided informed consent.

### Study protocol

All patients underwent unilateral contrast-enhanced MRI of metacarpophalangeal (MCP), wrist, and metatarsophalangeal (MTP) joints and musculoskeletal US at the same day < 2 weeks after first presentation. According to the protocol, imaging was done before disease-modifying anti-rheumatic drug (DMARD) initiation (including glucocorticoids) in patients with IA. DMARDs were not prescribed to patients with CSA. All patients were asked to stop NSAIDs 24 h before imaging. More details are provided supplementary.

### MR imaging and scoring

All patients were scanned on the same scanner (an MSK Extreme 1.5 T extremity MR system (GE Healthcare, Wisconsin, USA)). Unilateral MRI scans of wrist, MCP (2–5) and MTP (1–5) joints were made of the most affected side, or the dominant side in case of equally severe symptoms. Sequences acquired were coronal pre-contrast T1-weighted fast spin-echo (FSE) and coronal and axial post-contrast T1-weighted FSE with frequency-selective fat suppression of MCP and wrist, and post-contrast coronal and axial sequences of the MTP joints. More details are provided in reference and supplementary [17].

Each MRI-scan was scored according to RA MRI scoring (RAMRIS) method by two experienced readers (inter-reader intraclass correlation coefficients (ICC) > 0.94) [18, 19]. MRI scores for joints (synovitis) and tendons (tenosynovitis) ranged from 0 to 3. Mean scores of two readers were calculated and lesions were considered absent in case it was scored by only one reader.

### Musculoskeletal ultrasound scanning and scoring

A high-end US machine was used (GE Logiq E9, Genova, Italy) with a linear array transducer of 6–15 MHz. US examinations were performed bilaterally in GS and PD mode according to a standardised protocol. The same locations that were scanned by MRI were studied here. PD was assessed with a pulse repetition frequency of 0.8 kHz, and gain was set to a level until background signal was removed.

The presence of synovitis was assessed on a semi-quantitative scale (0–3) for GS/PD according to Szkudlarek et al. [20], and synovial effusion and hypertrophy were combined (called ‘modified Szkudlarek method’) [21]. Tenosynovitis was examined on a semi-quantitative scale (0–3) for GS/PD according to OMERACT [22]. A detailed US-scoring protocol is provided supplementary. All US scores per joint/tendon ranged from 0 to 3.

During the study, the newly developed EULAR-OMERACT-scoring method for synovitis was published [16]. To explore if the results changed when this definition was used, the static images of US were re-scored for GS synovitis by two examiners (ICC 0.92) and mean scores were calculated. The different scoring methods are described in Additional file 1: Table S1 and in the supplementary methods.

Imaging results were not communicated to clinicians at any time point.

### Statistical analyses

We compared semi-quantitative scores of US-detected synovitis and tenosynovitis to MRI-detected synovitis and tenosynovitis scores (each on a scale from 0 to 3), respectively, for each location using spearman’s correlation coefficients. For the primary analyses, we used the method according to the EULAR-OMERACT for GS synovitis. After analysing (semi-)quantitative data, US and MRI scores were dichotomized. For US, different cut-offs were studied: ≥ 1 and ≥ 2 for GS synovitis, ≥ 1 for PD synovitis and ≥ 1 for GS/PD tenosynovitis. Additionally, GS synovitis and tenosynovitis scores ≥ 2 or PD ≥ 1 were combined. MRI scores were dichotomized with ≥ 1 as cut-off and also on a cut-off based on findings from symptom-free volunteers, which has been published previously [23]. Then, an MRI was considered positive if synovitis or tenosynovitis was seen in < 5% of age-matched healthy controls. We calculated test characteristics for US with MRI as reference. Analyses were done on individual joint/tendon level and firstly presented on joint-group level (wrist, MCP, MTP joints) for reasons of clarity. Sub-analyses included stratification for patients presenting with IA and CSA and presentation of data at individual joint/tendon level. Finally, for GS synovitis, the ‘modified Szkudlarek method’ was compared to the EULAR-OMERACT method and also compared to MRI [16, 18, 21]. IBM SPSS (New York, USA) v23 was used.

## Results

### Study population

Seventy patients newly presenting to the rheumatology outpatient clinic (40 with recent-onset CSA, 30 with early IA) were included. Table 1 presents their baseline characteristics. The majority was female; mean age was 45 for patients with CSA and 57 for patients with IA (Additional file 1: Table S2). In total, 840 joints and 700 tendons were examined.

**Table 1:** Baseline characteristics of 70 patients studied

	All patients (*n* = 70)	
Age, mean (SD)	50	(15)
Female, *n* (%)	43	(61)
68-Tender joint count, median (IQR)	5	(2–8)
66-Swollen joint count, median (IQR)*	2	(1–6)
CRP (mg/L), median (IQR)	3	(3–11)
RF positive (≥ 3.5 IU/mL), *n* (%)	20	(29)
ACPA positive (≥ 7 U/mL), *n* (%)	16	(23)
Either RF or ACPA positive, *n* (%)	22	(31)

*Swollen joint count based on inflammatory arthritis (IA) patients, as all clinically suspect arthralgia (CSA) patients per definition do not have swollen joints

*ACPA* anti-citrullinated peptide antibody (anti-CCP2, EliA CCP, Phadia, the Netherlands, positive if ≥ 7 U/mL), *RF* immunoglobulin M-rheumatoid factor (positive if ≥ 3.5 IU/mL), *CRP* c-reactive protein (positive if ≥ 5 mg/L), *SD* standard deviation, *IQR* inter quartile range

### Synovitis detected by US versus MRI

**Fig. 1:**
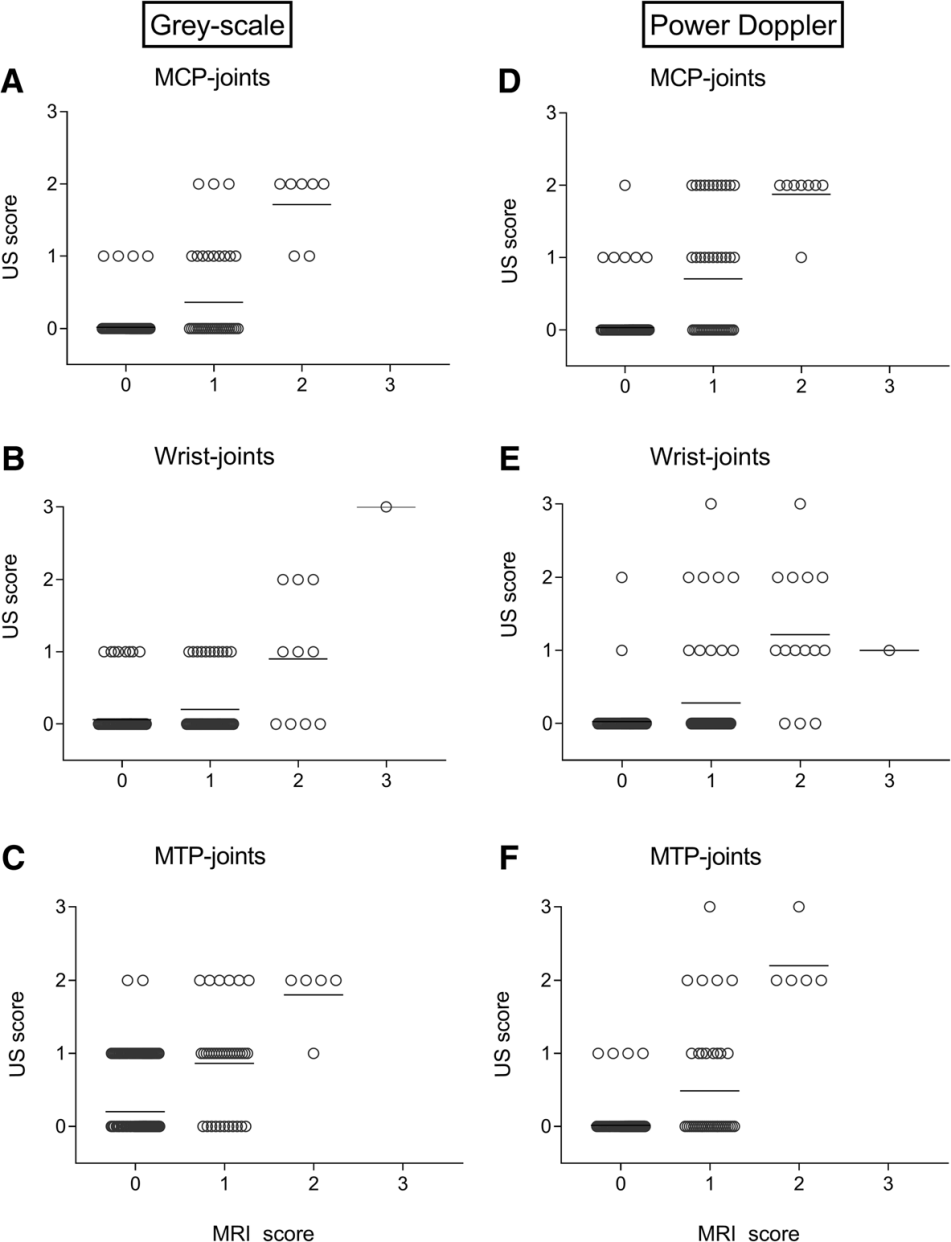
Greyscale ultrasound (according to EULAR-OMERACT definition, **a**–**c**) and power Doppler ultrasound-detected synovitis (**d**–**f**) versus MRI-detected synovitis on MCP, wrist and MTP joint level. Number of corresponding joints per MRI score was for **a** 0 = 222, 1 = 42, 2 = 7, 3 = 0; **b** 0 = 136, 1 = 55, 2 = 10, 3 = 1; **c** 0 = 285, 1 = 39, 2 = 5, 3 = 0; **d** 0 = 224, 1 = 48, 2 = 8, 3 = 0; **e** 0 = 137, 1 = 58, 2 = 14, 3 = 1; **f** 0 = 296, 1 = 39, 2 = 5, 3 = 0. Bars indicate the mean

Figure 1(a–c) presents the scores for GS-detected synovitis (EULAR-OMERACT method) versus MRI-detected synovitis (OMERACT-RAMRIS method). Analyses were performed on individual joints and tendons (i.e. MCP-2 of US versus MRI) and presented per joint group (MCPs, wrist, MTPs). All scores within joint groups were significantly correlated (Additional file 1: Table S3). In MTP joints, MRI scores of 0 infrequently coincided with scores of 1 for US (Fig. 1c); this is in contrast to findings on MCP and wrist level (Fig. 1a, b). In line with this observation, the corresponding test characteristics showed a high specificity (> 90%) for GS synovitis of wrist and MCP joints and a somewhat lower specificity of 80% for MTP joints. The sensitivity was poor for MCP and wrist (29–39%) and higher (75%) for MTP joints with MRI as reference (Table 2)

**Table 2:** Test characteristics for ultrasound-detected synovitis and tenosynovitis with MRI as reference

	Sensitivity	Specificity	AUC	Sensitivity	Specificity	AUC	Sensitivity	Specificity	AUC
Synovitis	GS ≥ 1 (EULAR-OMERACT)			PD ≥ 1			GS ≥ 2 (EULAR-OMERACT) or PD ≥ 1		
MCP joints	39 (27; 53)	98 (95; 99)	0.69	54 (41; 66)	97 (94; 99)	0.75	54 (41; 66)	97 (94; 99)	0.75
Wrist joints	29 (19; 40)	94 (89; 97)	0.61	30 (21; 41)	99 (95; 100)	0.64	30 (21; 41)	99 (95; 100)	0.64
MTP joints	75 (61; 85)	80 (75; 85)	0.78	41 (28; 56)	99 (97; 99)	0.70	68 (53; 80)	86 (81; 89)	0.77
Tenosynovitis	GS ≥ 1			PD ≥ 1			GS ≥ 2 or PD ≥ 1		
Extensor wrist tendons	78 (59; 86)	80 (74; 86)	0.78	58 (42; 73)	98 (95; 99)	0.78	67 (50; 80)	97 (93; 98)	0.82
Flexor wrist tendons	50 (31; 69)	94 (90; 97)	0.72	42 (24; 61)	99 (97; 100)	0.71	50 (31; 69)	99 (96; 100)	0.75
Flexor MCP tendons	74 (60; 84)	89 (84; 92)	0.81	19 (11; 31)	100 (98; 100)	0.59	36 (24; 49)	100 (98; 100)	0.68

Test characteristics are shown in percentages with a 95% CI except for the *AUC*, area under the receiver operating characteristic curve. *GS* greyscale, *PD* power Doppler

Subsequently, PD synovitis scores were compared to MRI. Also here, increased US scores were accompanied by increased MRI scores, and correlations were statistically significant (Additional file 1: Table S3). As presented (Fig. 1d–f), PD scores were only rarely ≥ 1 when MRI-detected synovitis scores were 0. Furthermore, we observed regularly that PD scores were 0 for joints that were scored ≥ 1 by MRI. These observations were reflected by the test characteristics, which showed a high specificity for PD (97–99%) for all locations (MTP, MCP, wrist) with only a low to moderate sensitivity (30–54%, Table 2).

Test characteristics when US positivity was defined by a combination of GS scores ≥ 2 or PD ≥ 1 are provided in Table 2. The combined scores showed a high specificity (> 92%) accompanied by an increased sensitivity for the MCP and wrist joints (30–54%) but not for the MTP joints (68%) in comparison to GS/PD alone.

### Tenosynovitis detected by US versus MRI

**Fig. 2:**
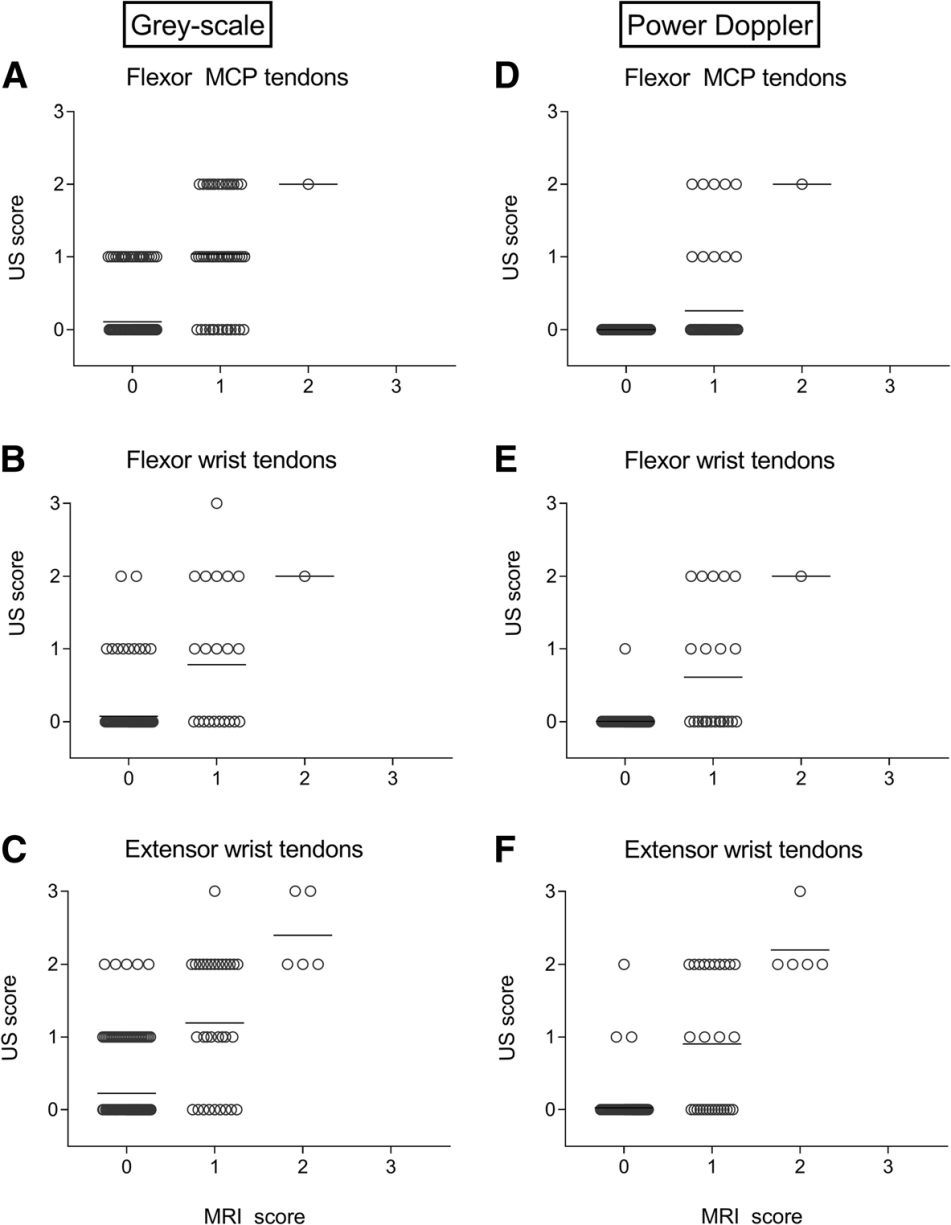
Greyscale (**a**–**c**) and power Doppler ultrasound-detected tenosynovitis (**d**–**f**) versus MRI-detected tenosynovitis of MCP flexor 2–5, wrist flexor and extensor tendons. Number of corresponding tendons per MRI score was for **a** 0 = 226, 1 = 52, 2 = 1, 3 = 0; **b** 0 = 186, 1 = 23, 2 = 1, 3 = 0; **c** 0 = 173, 1 = 32, 2 = 5, 3 = 0; **d** 0 = 226, 1 = 52, 2 = 1, 3 = 0; **e** 0 = 185, 1 = 23, 2 = 1, 3 = 0; **f** 0 = 171, 1 = 32, 2 = 5, 3 = 0. Bars indicate the mean

Figure 2(a–c) presents the data of GS-detected tenosynovitis versus MRI-detected tenosynovitis scores. MRI scores were significantly correlated to GS scores (Additional file 1: Table S3). However, scores ≥ 1 for MRI were also often accompanied by US scores of 0. Test characteristics were in line with these observations, with a specificity of 80% for the extensor wrist tendons and > 89% for the other tendons (flexor wrist, flexor MCPs), and a moderate sensitivity (50–78%, Table 2)

Figure 2(d–f) shows the data of PD tenosynovitis versus MRI. PD signals were infrequently increased. MRI detected 113 tendons with tenosynovitis (out of 700), while for PD this was only 45. The corresponding test characteristics in Table 2 showed a high specificity (98–100%) with a low to moderate sensitivity (19–58%)

Defining tenosynovitis as a combination of GS ≥ 2 or PD ≥ 1 slightly improved the test characteristics for the extensor and flexor tendons of the wrist, but not for the flexor tendons of the MCPs, compared to the separate ultrasound features (GS/PD) (Table 2).

### Cut-off for synovitis and tenosynovitis based on healthy volunteers

To investigate whether the excess of increased MRI-detected scores compared to US scores could be explained by the definition of positivity for MRI, we also applied a cut-off based on findings from symptom-free volunteers [23]. This resulted in a slightly increased sensitivity and AUC for GS-detected (teno)synovitis, while the specificity remained high compared to the main analyses. For PD, it only caused small differences (Additional file 1: Table S4).

### Sub-analyses stratified for IA and CSA

In Additional file 1: Figures S1–S4, we provided the data of the US synovitis and tenosynovitis scores (GS and PD) versus MRI for patients with CSA and IA separately. As expected, synovitis and tenosynovitis were less frequently present and/or less severe in patients with CSA than IA. However, the pattern of concordance between MRI and US was similar. We also calculated test characteristics for patients with CSA and IA separately (Additional file 1: Table S5). The sensitivity for US with MRI as reference was lower in CSA than in patients with IA. The specificity was similar in both populations.

### Data presented on individual joint/tendon level

For clarity, the main results were presented on joint-group level, although analyses were performed on joint/tendon level. However, as findings on different joints/tendons might be different and these differences cannot be seen by presentation the joint-group level, we also provided test characteristics for each joint/tendon separately (Additional file 1: Tables S6, S7). In general, results were similar with a low to moderate sensitivity and high specificity. Remarkably, for the flexor tendons of the wrist (GS), all flexors had high specificity (88–100%). However, the sensitivity varied broadly: 71% for the FPL, 55% for the FCR and only 17% for the FDS/FDP. Also for PD, the sensitivity for tenosynovitis was generally low (17–64%).

**Fig. 3:**
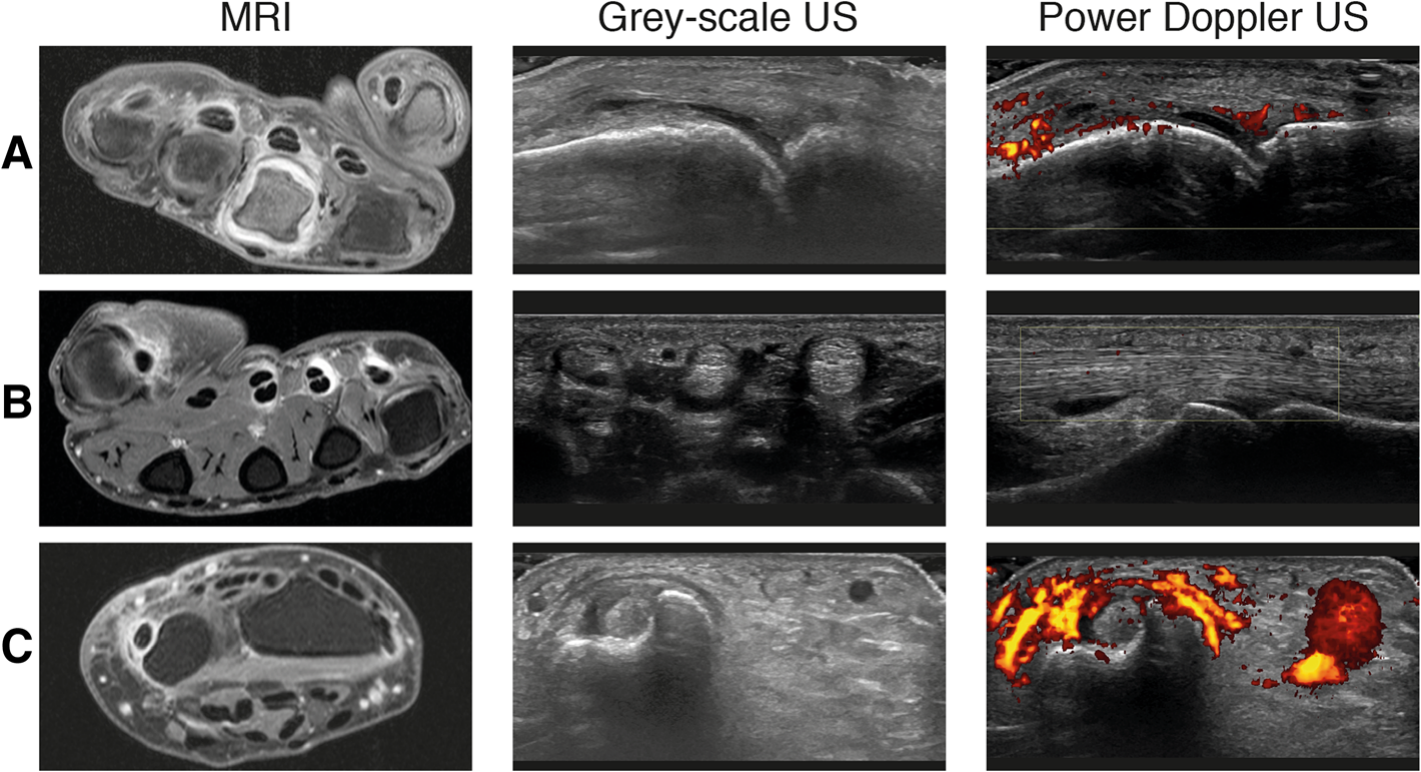
Examples of MRI-detected synovitis and tenosynovitis with corresponding greyscale and power Doppler ultrasound images. Examples of inflammation identified by MRI that were verified through ultrasound (US). Even though sometimes more inflammation was present on the MRI scan, we choose to show the corresponding US images of only one joint or tendon that was present on the image. **a** An example of synovitis MCP-3 by MRI, which was confirmed by greyscale (GS) and power Doppler (PD) US images of the same joint. **b** Tenosynovitis of the flexor of MCP-4 which was confirmed by GS but not by PD on US. **c** Inflammation of the extensor carpi ulnaris tendon at the wrist level which was confirmed by both GS and PD on US

Examples of MRI-detected (teno)synovitis versus GS/PD are illustrated by Fig. 3.

### Evaluation of two scoring methods for GS-detected synovitis

Due to recent advances in scoring methods for GS, two methods were applied and test characteristics were also determined for GS by the modified Szkudlarek method [21] with MRI as reference (Table 3). The modified Szkudlarek method had a higher sensitivity of 68–91% and lower specificity of 52–71% than the EULAR-OMERACT method (sensitivity 29–75%, specificity 80–98%) compared to MRI. Thereafter, we compared the scores of the two scoring methods for GS for each joint. The modified Szkudlarek method generally had higher scores than the EULAR-OMERACT method (see Additional file 1: Table S8–S10).

**Table 3:** Test characteristics for greyscale ultrasound-detected synovitis with MRI as a reference for the ‘old’ synovitis definition according to the modified Szkudlarek method and for the ‘new’ EULAR-OMERACT synovitis definition

	Sensitivity	Specificity	AUC	Sensitivity	Specificity	AUC
Synovitis	GS ≥ 1 (modified Szkudlarek)			GS ≥ 2 (modified Szkudlarek)		
MCP joints	73 (60; 83)	70 (64; 76)	0.72	39 (28; 52)	96 (93; 98)	0.68
Wrist joints	68 (57; 78)	71 (63; 78)	0.70	34 (24; 46)	97 (93; 99)	0.66
MTP joints	91 (79; 96)	52 (47; 58)	0.72	64 (49; 76)	92 (88; 94)	0.78
Synovitis	GS ≥ 1 (EULAR-OMERACT)			GS ≥ 2 (EULAR-OMERACT)		
MCP joints	39 (27; 53)	98 (95; 99)	0.69	17 (9; 30)	100 (98; 100)	0.58
Wrist joints	29 (19; 40)	94 (89; 97)	0.61	9 (4; 17)	100 (97; 100)	0.54
MTP joints	75 (61; 85)	80 (75; 85)	0.78	23 (13; 37)	99 (97; 100)	0.61

Test characteristics are shown in percentages with a 95% CI except for the *AUC*, area under the receiver operating characteristic curve. *GS* greyscale

## Discussion

This large cross-sectional study compared US and MRI findings of synovitis and tenosynovitis on the joint and tendon levels, respectively, in patients newly presenting with early IA and CSA. These are the populations where imaging modalities can have a specific role in the diagnostic process. The newly developed EULAR-OMERACT-scoring method for GS-detected synovitis for US was used. Our data showed that US findings were highly specific and rarely ‘false-positive’, but also less sensitive compared to MRI, resulting in ‘false-negative results’. This suggests that MRI cannot be replaced by US while maintaining its sensitivity on the level of joints and tendons. How this affects the predictive accuracy needs to be investigated further in longitudinal studies.

Two different scoring methods for GS-detected synovitis were applied: the EULAR-OMERACT method and the modified Szkudlarek method, which combines synovial effusion and hypertrophy [16, 21]. Direct comparison of both scoring methods for GS synovitis showed that higher scores were obtained by the modified Szkudlarek method. In line with this and compared to MRI, the modified Szkudlarek method had more false positives which resulted in a higher sensitivity but lower specificity than the EULAR-OMERACT method. The false-positive results (MRI scores 0, GSUS > 0) obtained by the modified Szkudlarek method might be explained by the fact that it evaluates a combination of synovial effusion and hypertrophy, while in the recent EULAR-OMERACT definition hypertrophy regardless of the presence of synovial effusion was evaluated [16], and the fact that contrast-enhanced MRI also does not visualise joint effusion. Thus, although this study did not primarily aim to compare the ‘old’ and ‘new’ GS synovitis scores, present data also showed the relationship between both GS scoring methods and revealed that the EULAR-OMERACT synovitis score for US was more concordant to the OMERACT-RAMRIS method for MRI.

Unfortunately, the definition of the EULAR-OMERACT for GS synovitis was published when this study had already started [16]. Consequently, synovitis had already been scored according to the modified Szkudlarek method. Therefore, static US images were rescored according to the EULAR-OMERACT method, which might be a potential limitation, as scoring of static images can be challenging. We used two independent readers to assess the static images; both readers showed excellent agreement between the reading results, which supports the reliability of these data.

Since the role of synovial effusion in the pathologic process of RA and other types of IA is not yet fully understood, synovial effusion was not explicitly taken into account, except within the modified Szkudlarek method [21]. Synovial effusion often has been detected in healthy persons by US, especially in the feet [24]. Unfortunately, up to now, age-related normal values for US-detected pathologies such as synovial effusion, synovial hypertrophy, tenosynovitis and erosions are still unknown and should be subject for future studies. Furthermore, it would be interesting to see the effect of findings in healthy symptom-free individuals for the definition of positivity for US. This is also subject for future research.

Importantly, there were differences between the scoring methods for US and MRI. All scoring methods consisted of semi-quantitative scales ranging from 0 to 3. However, the requirements for each grade were different for US and MRI (Additional file 1: Table S1). Thus, different definitions for the different scoring methods hamper direct comparison of the different grades, though as presented by Figs. 1 and 2, increased US scores generally coincided with increased MRI scores. To assess whether this was similar in patients with CSA and IA, we also repeated the analyses for both populations separately. In both populations, higher US scores were present in patients with higher MRI scores (Additional file 1: Figures S1–S4). However, the test characteristics were not completely similar. Although the specificity for US was similar in both populations, the sensitivity was lower in patients with CSA compared to IA. CSA patients have less severe inflammation than patients with IA and current data implied that in this setting of subclinical inflammation, US is less sensitive than MRI.

Another issue is the cut-off used for dichotomization. Our US cut-offs are frequently used in the literature. For GS, we observed that increasing the cut-off from ≥ 1 to ≥ 2 resulted in an increased specificity and a notably decreased sensitivity. This phenomenon is often observed when changing cut-offs. Based on AUCs, a cut-off ≥ 1 could be considered more favourably than ≥ 2. Also, the cut-off for MRI positivity was explored. In addition to using a cut-off of mean ≥ 1, we applied a cut-off based on healthy volunteers [23]. This caused only minor improvements in the test characteristics for US compared to MRI.

A strength of this study was that besides synovitis, also tenosynovitis was evaluated; this imaging feature is less often studied than synovitis while it is important, as tenosynovitis in IA and CSA has been shown predictive of RA development, both in studies that used MRI [9, 25] and US [7]. Furthermore, this study examined patients at risk for RA and applied the new EULAR-OMERACT score for GS-detected synovitis. We also did not only examine the wrist and MCP, but also the MTP joints. In contrast, a recent meta-analysis compared the accuracy of US-detected synovitis versus MRI in wrist, MCP, PIP, and knee joints, but not MTP joints in patients with classified RA [26]. The included studies were also not scored according to the EULAR-OMERACT method. Despite these differences, the sensitivity and specificity for GS/PD-detected synovitis observed in this study compared to our data are roughly similar. Also GS tenosynovitis was previously studied by Wakefield et al. in MCP joints of classified RA-patients and were comparable to our results from patients in earlier disease phases, showing a high specificity and moderate sensitivity [11].

In our data on tenosynovitis, the sensitivity was particularly low for the FDS/FDP tendon. A possible explanation could be that this tendon is located below the retinaculum flexorum, deeper in the wrist tissue than other tendons. Also, PDUS tenosynovitis had only a low to moderate sensitivity, despite the use of high-end US machine with a sensitive power Doppler. PD-detected tenosynovitis had only a small or no additive value to GS tenosynovitis, particularly for the MCP-flexor tendons. A reason for this could be that PD performs better from the dorsal side of the joint than from the palmar side, which may have contributed to this finding [16, 27]. Although replication in other studies is needed, the current data with MR as reference suggests that PDUS-detected tenosynovitis had no clear additive value to GSUS, which is in contrast to findings for synovitis.

This cross-sectional study is the first that examined the concordance between synovitis detected by US and MRI in the feet of patients with (suspicion on imminent) early RA. Interestingly, GS synovitis had a higher sensitivity in the feet than in the hand joints, which was at the cost of a lower specificity (implying a higher frequency of false-positive signals in MTP joints).

MRI was the reference in this cross-sectional study on the joint/tendon level, showing false-negative findings for synovitis and tenosynovitis. For clinical purposes, analyses on patient level are also relevant, as patients often have > 1 joint affected and at least 1 joint with subclinical inflammation might be considered sufficient to indicate disease. Analyses on the patient level showed that US missed only 1/44 patients (GS) and 14/44 (PD) compared to MRI (cut-offs ≥ 1, data not shown). Hence, there is less discordance on the patient level than on the joint/tendon level. The comparability of US and MRI to accurately predict RA development remains an outstanding question, for which longitudinal studies with RA development as outcome are needed.

In conclusion, this is the first study that used the recently developed EULAR-OMERACT method for US in comparison to MRI, in patients consecutively presenting with early IA and CSA. These are the populations in which these imaging modalities can be used to detect (imminent) RA. US had a good specificity, but was less sensitive compared to MRI on the local tendon and joint level. However, US is more easily available, less time-consuming and has lower costs than MRI. Longitudinal studies in ‘at-risk’ populations are needed to directly compare the predictive accuracy of MRI and US while using up-to-date scoring methods.

## Additional file

Additional file 1: **Table S1.** Different greyscale ultrasound scoring methods. **Table S2.** Baseline characteristics of 70 patients studied. **Table S3.** Correlation coefficients of US versus MRI for the different locations. **Table S4.** Test characteristics for ultrasound-detected synovitis and tenosynovitis with MRI as reference, cut-off for positivity for MRI based on healthy controls. **Table S5.** Test characteristics for US-detected synovitis and tenosynovitis with MRI as reference for CSA and IA separately. **Table S6.** Test characteristics per joint for greyscale (EULAR OMERACT definition) and power Doppler ultrasound-detected synovitis with MRI as a reference. **Table S7.** Test characteristics per joint for greyscale and power Doppler ultrasound-detected tenosynovitis with MRI as a reference. **Table S8.** Greyscale ultrasound detected synovitis according to EULAR-OMERACT definition versus Szkudlarek on MCP joint level. **Table S9.** Greyscale ultrasound detected synovitis (according to EULAR-OMERACT definition) versus greyscale ultrasound detected synovitis (according to Szkudlarek) on wrist joint level. **Table S10.** Greyscale ultrasound detected synovitis (according to EULAR-OMERACT definition) versus greyscale ultrasound detected synovitis (according to Szkudlarek) on MCP joint level. **Figure S1.** Grey-scale (according to EULAR-OMERACT definition; A,B,C) and power Doppler ultrasound-detected synovitis (D,E,F) versus MRI-detected synovitis on MCP, wrist and MTP joint level for IA. **Figure S2.** Greyscale (according to EULAR-OMERACT definition; A,B,C) and power Doppler ultrasound-detected synovitis (D,E,F) versus MRI-detected synovitis on MCP, wrist and MTP joint level for CSA. **Figure S3.** Greyscale (A,B,C) and power Doppler ultrasound-detected tenosynovitis (D,E,F) versus MRI-detected tenosynovitis of MCP flexor 2–5, wrist flexor and extensor tendons for IA. **Figure S4.** Greyscale (A,B,C) and power Doppler ultrasound-detected tenosynovitis (D,E,F) versus MRI-detected tenosynovitis of MCP flexor 2–5, wrist flexor and extensor tendons for CSA. (DOCX 986 kb)

## Abbreviations

CSA: Clinically suspect arthralgia
DMARD: Disease-modifying anti-rheumatic drug
EAC: Early arthritis clinic
EULAR: European League Against Rheumatism
FCR: Flexor carpi radialis
FDS/FDP: Flexor digitorum superficialis/profundus
FPL: Flexor pollicis longus
GS: Greyscale
IA: Inflammatory arthritis
ICC: Intraclass correlation coefficients
MCP: Metacarpophalangeal
MRI: Magnetic resonance imaging
MTP: Metatarsophalangeal
NSAID: Non-steroid anti-inflammatory drug
OMERACT: Outcome Measures in Rheumatology Clinical Trials
PD: Power Doppler
RA: Rheumatoid arthritis
RAMRIS: Rheumatoid arthritis MRI scoring
US: Musculoskeletal ultrasound
