# Supplementary material for: Do musculoskeletal ultrasound and magnetic resonance imaging identify synovitis and tenosynovitis at the same joints and tendons? A comparative study in early inflammatory arthritis and clinically suspect arthralgia
Source: Arthritis Res Ther. 2019 Feb 14;21:59. doi: 10.1186/s13075-019-1824-z (PMC6376767; doi:10.1186/s13075-019-1824-z)
Supplement: Supplementary file 1 — Table S1. Different greyscale ultrasound scoring methods. Table S2. Baseline characteristics of 70 patients studied. Table S3. Correlation coefficients of US versus MRI for the different locations. Table S4. Test characteristics for ultrasound-detected synovitis and tenosynovitis with MRI as reference, cut-off for positivity for MRI based on healthy controls. Table S5. Test characteristics for US-detected synovitis and tenosynovitis with MRI as reference for CSA and IA separately. Table S6. Test characteristics per joint for greyscale (EULAR OMERACT definition) and power Doppler ultrasound-detected synovitis with MRI as a reference. Table S7. Test characteristics per joint for greyscale and power Doppler ultrasound-detected tenosynovitis with MRI as a reference. Table S8. Greyscale ultrasound detected synovitis according to EULAR-OMERACT definition versus Szkudlarek on MCP joint level. Table S9. Greyscale ultrasound detected synovitis (according to EULAR-OMERACT definition) versus greyscale ultrasound detected synovitis (according to Szkudlarek) on wrist joint level. Table S10. Greyscale ultrasound detected synovitis (according to EULAR-OMERACT definition) versus greyscale ultrasound detected synovitis (according to Szkudlarek) on MCP joint level. Figure S1. Grey-scale (according to EULAR-OMERACT definition; A,B,C) and power Doppler ultrasound-detected synovitis (D,E,F) versus MRI-detected synovitis on MCP, wrist and MTP joint level for IA. Figure S2. Greyscale (according to EULAR-OMERACT definition; A,B,C) and power Doppler ultrasound-detected synovitis (D,E,F) versus MRI-detected synovitis on MCP, wrist and MTP joint level for CSA. Figure S3. Greyscale (A,B,C) and power Doppler ultrasound-detected tenosynovitis (D,E,F) versus MRI-detected tenosynovitis of MCP flexor 2–5, wrist flexor and extensor tendons for IA. Figure S4. Greyscale (A,B,C) and power Doppler ultrasound-detected tenosynovitis (D,E,F) versus MRI-detected tenosynovitis of MCP flexor 2–5, wrist flexor [file 13075_2019_1824_MOESM1_ESM.docx]

**SUPPLEMENTARY DATA**

TABLES

Table S1. Different Grey-scale ultrasound scoring methods.

|  | **GS score for joint effusion**[1] | **Szkudlarek GS synovitis score**[1] | **Modified Szkudlarek GS synovitis score**[2] | **EULAR-OMERACT GS synovitis score**[3] |
| --- | --- | --- | --- | --- |
| Grade 0 | No effusion | No synovial thickening | No synovial hypertrophy or effusion | No synovial hypertrophy, regardless of presence of effusion |
| Grade 1 | Minimal amount of fluid | Minimal synovial thickening  -filling the angle between the peri-articular bones, without bulging over the line, linking tops of the bones | Minimal effusion and/or hypertrophy,  -filling the angle between the peri-articular bones, without bulging over the line, linking tops of the bones | Synovial hypertrophy with or without effusion  -up to level of horizontal line that connects bone surfaces |
| Grade 2 | Moderate amount of fluid, without distension of the joint capsule | Synovial thickening  -bulging over the line linking tops of the peri-articular bones but without extension along the bone diaphysis | Moderate effusion and/or hypertrophy  -bulging over the line linking tops of the peri-articular bones but without extension along the bone diaphysis | Synovial hypertrophy with or without effusion  -extending beyond joint line but with upper surface convex or hypertrophy extending beyond joint line but with upper surface flat |
| Grade 3 | Extensive amount of fluid, with distension of the joint capsule | Synovial thickening  -bulging over the line linking tops of the periarticular bones and with extension to at least one of the bone diaphyses | Extensive effusion and/or hypertrophy  -bulging over the line linking tops of the periarticular bones and with extension to at least one of the bone diaphyses | Synovial hypertrophy  - with or without effusion extending beyond joint line but with upper surface flat or convex |

**Table S2**. Baseline characteristics of 70 patients studied.

|  | **IA patients**  **(n=30)** | | **CSA patients**  **(n=40)** | |
| --- | --- | --- | --- | --- |
| Age, mean (SD) | 57 | (16) | 45 | (11) |
| Female, n (%) | 15 | (50) | 28 | (70) |
| 68-Tender joint count, median (IQR) | 5 | (2-7) | 5 | (3-10) |
| 66-Swollen joint count, median (IQR) | 2 | (1-6) | - |  |
| CRP (mg/L), median (IQR) | 8 | (3-18) | 3 | (3-7) |
| RF positive (≥3.5 IU/mL), n (%) | 7 | (23) | 13 | (33) |
| ACPA positive (≥7 U/mL), n (%) | 7 | (23) | 9 | (23) |
| Either RF or ACPA positive, n (%) | 8 | (27) | 14 | (35) |

ACPA, anti-citrullinated peptide antibody (anti-CCP2, EliA CCP, Phadia, the Netherlands, positive if ≥7 U/mL); RF, immunoglobulin M-rheumatoid factor (RF) (positive if ≥3.5 IU/mL); CRP, c-reactive protein (positive if ≥5mg/L); SD, standard deviation; IQR, Inter quartile range.

Table S3. Correlation coefficients of US versus MRI for the different locations.

|  | **MRI versus GSUS** | | **MRI versus PDUS** | |
| --- | --- | --- | --- | --- |
|  | Spearman’s rho | p-value | Spearman’s rho | p-value |
| *Synovitis* |  |  |  |  |
| MCP joints | 0.57 | <0.001 | 0.64 | <0.001 |
| MTP joints | 0.43 | <0.001 | 0.53 | <0.001 |
| Wrist joints | 0.22 | 0.002 | 0.40 | <0.001 |
|  |  |  |  |  |
| *Tenosynovitis* |  |  |  |  |
| Flexor MCP tendons | 0.62 | <0.001 | 0.43 | <0.001 |
| Flexor Wrist tendons | 0.46 | <0.001 | 0.59 | <0.001 |
| Extensor Wrist tendons | 0.54 | <0.001 | 0.70 | <0.001 |
|  |  |  |  |  |

Correlation coefficients of two different semi-quantitative scoring methods. Obtained values are therefore not representative of the exact concordance as scores of US and MRI have different requirements. GSUS-synovitis is according to EULAR-OMERACT definition.

Table S4. Test characteristics for ultrasound-detected synovitis and tenosynovitis with MRI as reference, cut-off for positivity for MRI based on healthy controls.

|  | **Sensitivity** | **Specificity** | **AUC** | **Sensitivity** | **Specificity** | **AUC** |
| --- | --- | --- | --- | --- | --- | --- |
| *Synovitis* | *GS ≥1 (EULAR-OMERACT)* | | | *PD ≥1* | | |
| MCP joints | 47  (27; 68) | 94  (90; 96) | 0.71 | 55  (34; 74) | 90  (86; 93) | 0.73 |
| Wrist joints | 52  (32; 72) | 91  (86; 94) | 0.71 | 64  (43; 80) | 95  (90; 97) | 0.79 |
| MTP joints | 72  (55; 84) | 78  (73; 82) | 0.75 | 38  (23; 55) | 97  (94; 98) | 0.67 |
| *Tenosynovitis* | *GS ≥1* | |  | *PD ≥1* | | |
| Extensor wrist tendons | 80  (63; 90) | 79  (72; 84) | 0.79 | 57  (39; 73) | 96  (92; 98) | 0.76 |
| Flexor wrist tendons | 50  (31; 69) | 94  (90; 97) | 0.72 | 42  (24; 61) | 99  (97; 100) | 0.71 |
| Flexor MCP tendons | 54  (35; 71) | 80  (75; 85) | 0.67 | 19  (9; 38) | 98  (95; 99) | 0.59 |

Test characteristics are shown in percentages with a 95% CI except for the AUC, area under the receiver operating characteristic curve; GS: Grey-scale Ultrasound; PD: Power Doppler.

Table S5. Test characteristics for US-detected synovitis and tenosynovitis with MRI as reference for CSA and IA separately.

| *IA-patients* | **Sensitivity** | **Specificity** | **AUC** | **Sensitivity** | **Specificity** | **AUC** |
| --- | --- | --- | --- | --- | --- | --- |
| *Synovitis* | *GS ≥1 (EULAR-OMERACT)* | | | *PD ≥1* | | |
| MCP joints | 57  (41; 71) | 95  (88; 98) | 0.76 | 63  (48; 76) | 97  (91; 99) | 0.80 |
| Wrist joints | 36  (24; 50) | 90  (77; 96) | 0.63 | 36  (24; 50) | 98  (87; 100) | 0.67 |
| MTP joints | 87  (71; 95) | 76  (67; 83) | 0.82 | 42  (26; 59) | 99  (95; 100) | 0.71 |
| *Tenosynovitis* | *GS ≥1* | |  | *PD ≥1* | | |
| Extensor wrist tendons | 82  (64; 92) | 80  (69; 88) | 0.82 | 64  (46; 79) | 98  (91; 100) | 0.81 |
| Flexor wrist tendons | 55  (35; 73) | 88  (78; 94) | 0.72 | 45  (27; 65) | 99  (92; 100) | 0.72 |
| Flexor MCP tendons | 84  (69; 92) | 89  (81; 94) | 0.87 | 22  (11; 37) | 100  (96; 100) | 0.61 |
| *CSA-patients* |  |  |  |  |  |  |
| *Synovitis* | *GS ≥1 (EULAR-OMERACT)* | | | *PD ≥1* | | |
| MCP joints | 0  (0; 20) | 100  (97; 100) | 0.50 | 27  (11; 52) | 97  (93; 99) | 0.62 |
| Wrist joints | 13  (5; 32) | 96  (90; 98) | 0.55 | 17  (7; 37) | 99  (94; 100) | 0.58 |
| MTP joints | 46  (23; 71) | 83  (77; 88) | 0.65 | 38  (18; 64) | 98  (95; 99) | 0.68 |
| *Tenosynovitis* | *GS ≥1* | |  | *PD ≥1* | | |
| Extensor wrist tendons | 50  (22; 78) | 80  (72; 87) | 0.65 | 38  (14; 69) | 98  (94; 100) | 0.68 |
| Flexor wrist tendons | 0  (0; 66) | 97  (93; 99) | 0.49 | 0  (0; 66) | 100  (97; 100) | 0.50 |
| Flexor MCP tendons | 50  (28; 72) | 89  (83; 93) | 0.69 | 13  (3; 36) | 100  (97; 100) | 0.56 |

Table S6. Test characteristics per joint for Grey-scale (EULAR OMERACT definition) and power Doppler Ultrasound-detected synovitis with MRI as a reference.

|  | **Sensitivity** | **Specificity** | **AUC** | **Sensitivity** | **Specificity** | **AUC** |
| --- | --- | --- | --- | --- | --- | --- |
| *Synovitis* | *GS ≥1 (EULAR OMERACT)* | |  | *Synovitis PD ≥1* | | |
| **MCP joints** | | |  |  | | |
| MCP 2 | 50  (29; 71) | 100  (93; 100) | 0.75 | 63  (41; 81) | 96  (87; 99) | 0.77 |
| MCP 3 | 40  (20; 64) | 96  (87; 99) | 0.68 | 56  (33; 77) | 96  (87; 99) | 0.76 |
| MCP 4 | 20  (6; 51) | 97  (88; 99) | 0.58 | 36  (15; 65) | 97  (88; 99) | 0.67 |
| MCP 5 | 38  (14; 69) | 100  (94; 100) | 0.69 | 50  (24; 76) | 100  (94; 100) | 0.75 |
| **Wrist joints** | |  |  |  |  |  |
| Radio-ulnar | 16  (6; 38) | 94  (84; 98) | 0.55 | 25  (11; 47) | 98  (90; 100) | 0.62 |
| Radio-carpal | 38  (22; 57) | 90  (78; 96) | 0.65 | 30  (16; 48) | 98  (88; 100) | 0.64 |
| Inter-carpal | 28  (14; 48) | 98  (88; 100) | 0.63 | 35  (19; 54) | 100  (92; 100) | 0.67 |
| **MTP joints** | |  |  |  |  |  |
| MTP 1 | 88  (64; 97) | 62  (48; 74) | 0.75 | 50  (28; 72) | 98  (90; 100) | 0.74 |
| MTP 2 | 70  (40; 89) | 66  (53; 77) | 0.68 | 20  (6; 51) | 100  (94; 100) | 0.60 |
| MTP 3 | 80  (38; 96) | 82  (71; 90) | 0.81 | 40  (12; 77) | 100  (94; 100) | 0.70 |
| MTP 4 | 100  (51; 100) | 93  (84; 97) | 0.97 | 50  (15; 85) | 100  (94; 100) | 0.75 |
| MTP 5 | 44  (19; 73) | 95  (86; 98) | 0.70 | 44  (19; 73) | 95  (86; 98) | 0.70 |

Test characteristics are shown in percentages with a 95% CI except for the AUC, area under the receiver operating characteristic curve.

GS: Grey-scale; PD: Power Doppler; MCP: metacarpophalangeal; MTP: metatarsophalangeal.

Table S7. Test characteristics per joint for Grey-scale and power Doppler Ultrasound-detected tenosynovitis with MRI as a reference.

|  | **Sensitivity** | **Specificity** | **AUC** | **Sensitivity** | **Specificity** | **AUC** |
| --- | --- | --- | --- | --- | --- | --- |
| *Tenosynovitis* | *GS ≥1* | |  | *Tenosynovitis PD ≥1* | | |
| **MCP flexor tendons** | | |  |  | | |
| FD MCP 2 | 88  (64; 97) | 89  (78; 95) | 0.88 | 31  (14; 56) | 100  (93; 100) | 0.66 |
| FD MCP 3 | 46  (23; 71) | 95  (86; 98) | 0.70 | 15  (4; 42) | 100  (94; 100) | 0.58 |
| FD MCP 4 | 91  (62; 98) | 93  (84; 97) | 0.92 | 18  (5; 48) | 100  (94; 100) | 0.59 |
| FD MCP 5 | 69  (42; 87) | 79  (67; 88) | 0.74 | 8  (1; 33) | 100  (94; 100) | 0.54 |
| **Flexor wrist tendons** | |  |  |  |  |  |
| FDS/FDP | 17  (3; 56) | 100  (94; 100) | 0.58 | 17  (3; 56) | 100  (94; 100) | 0.58 |
| FPL | 71  (36; 92) | 94  (85; 98) | 0.83 | 29  (8; 64) | 100  (94; 100) | 0.64 |
| FCR | 55  (28; 79) | 88  (77; 94) | 0.71 | 64  (35; 85) | 98  (91; 100) | 0.81 |
| **Extensor wrist tendons** | |  |  |  |  |  |
| ECR | 78  (45; 94) | 78  (66; 87) | 0.78 | 44  (19; 73) | 100  (94; 100) | 0.72 |
| EDC/IP | 70  (40; 89) | 83  (72; 91) | 0.77 | 50  (24; 76) | 100  (94; 100) | 0.75 |
| ECU | 76  (53; 90) | 79  (67; 88) | 0.78 | 71  (47; 87) | 94  (85; 98) | 0.82 |

Test characteristics are shown in percentages with a 95% CI except for the AUC, area under the receiver operating characteristic curve.

GS: Grey-scale; PD: Power Doppler; Extensor carpi radialis longus et brevis: ECR; Extensor digitorum communis and indices proprius: EDC/IP; Extensor carpi ulnaris: ECU; Flexor carpi radialis: FCR; Flexor pollicis longus: FPL; Flexor digitorum superficialis and profundus: FDS/FDP; flexor digitorum (FD) tendons 2-5 on the MCP-level.

Table S8. Grey-scale Ultrasound detected synovitis according to EULAR-OMERACT definition versus Szkudlarek on MCP joint level.

| **MCP 2** |  | GS according to Szkudlarek | | | | |
| --- | --- | --- | --- | --- | --- | --- |
|  | | **0** | **1** | **2** | | **3** |
| GS according to EULAR-OMERACT | |  |  |  | |  |
|  | **0** | 31 | 24 | 5 | | 0 |
|  | **1** | 0 | 3 | 5 | | 0 |
|  | **2** | 0 | 0 | 0 | | 1 |
|  | **3** | 0 | 0 | 0 | | 0 |
| **MCP 3** |  | GS according to Szkudlarek | | | | |
|  | | **0** | **1** | **2** | | **3** |
| GS according to EULAR-OMERACT | |  |  |  | |  |
|  | **0** | 40 | 17 | 4 | | 0 |
|  | **1** | 0 | 2 | 1 | | 1 |
|  | **2** | 0 | 0 | 2 | | 2 |
|  | **3** | 0 | 0 | 0 | | 0 |
| **MCP 4** |  | GS according to Szkudlarek | | | | |
|  | | **0** | **1** | | **2** | **3** |
| GS according to EULAR-OMERACT | |  |  | |  |  |
|  | **0** | 50 | 14 | | 1 | 0 |
|  | **1** | 1 | 0 | | 1 | 0 |
|  | **2** | 0 | 0 | | 1 | 1 |
|  | **3** | 0 | 0 | | 0 | 0 |
| **MCP 5** |  | GS according to Szkudlarek | | | | |
|  | | **0** | **1** | | **2** | **3** |
| GS according to EULAR-OMERACT | |  |  | |  |  |
|  | **0** | 49 | 13 | | 3 | 0 |
|  | **1** | 0 | 1 | | 1 | 0 |
|  | **2** | 0 | 0 | | 0 | 2 |
|  | **3** | 0 | 0 | | 0 | 0 |

Number of patients for each joint score according to Szkudlarek versus the EULAR/OMERACT revised score. Total number of patients was 70, but rarely a score was missing.

GS: Grey-scale.Table S9. Grey-scale Ultrasound detected synovitis (according to EULAR-OMERACT definition) versus Grey-scale Ultrasound detected synovitis (according to Szkudlarek) on wrist joint level.

| **Radio-Ulnar** |  | GS according to Szkudlarek | | | | |
| --- | --- | --- | --- | --- | --- | --- |
|  | | **0** | **1** | **2** | | **3** |
| GS according to EULAR-OMERACT | |  |  |  | |  |
|  | **0** | 53 | 10 | 0 | | 0 |
|  | **1** | 0 | 3 | 1 | | 0 |
|  | **2** | 0 | 0 | 2 | | 0 |
|  | **3** | 0 | 0 | 0 | | 0 |
| **Radio-Carpal** |  | GS according to Szkudlarek | | | | |
|  | | **0** | **1** | **2** | | **3** |
| GS according to EULAR-OMERACT | |  |  |  | |  |
|  | **0** | 21 | 28 | 6 | | 0 |
|  | **1** | 0 | 5 | 7 | | 0 |
|  | **2** | 0 | 0 | 1 | | 0 |
|  | **3** | 0 | 0 | 0 | | 1 |
| **Inter-Carpal** |  | GS according to Szkudlarek | | | | |
|  | | **0** | **1** | | **2** | **3** |
| GS according to EULAR-OMERACT | |  |  | |  |  |
|  | **0** | 45 | 14 | | 2 | 0 |
|  | **1** | 0 | 1 | | 4 | 1 |
|  | **2** | 0 | 0 | | 0 | 2 |
|  | **3** | 0 | 0 | | 0 | 0 |

Table S10. Grey-scale Ultrasound detected synovitis (according to EULAR-OMERACT definition) versus Grey-scale Ultrasound detected synovitis (according to Szkudlarek) on MCP joint level.

| **MTP 1** |  | GS according to Szkudlarek | | | | |
| --- | --- | --- | --- | --- | --- | --- |
|  | | **0** | **1** | **2** | | **3** |
| GS according to EULAR-OMERACT | |  |  |  | |  |
|  | **0** | 18 | 16 | 0 | | 0 |
|  | **1** | 0 | 18 | 7 | | 0 |
|  | **2** | 0 | 0 | 8 | | 0 |
|  | **3** | 0 | 0 | 0 | | 0 |
| **MTP 2** |  | GS according to Szkudlarek | | | | |
|  | | **0** | **1** | **2** | | **3** |
| GS according to EULAR-OMERACT | |  |  |  | |  |
|  | **0** | 12 | 28 | 1 | | 0 |
|  | **1** | 0 | 13 | 9 | | 1 |
|  | **2** | 0 | 0 | 2 | | 1 |
|  | **3** | 0 | 0 | 0 | | 0 |
| **MTP 3** |  | GS according to Szkudlarek | | | | |
|  | | **0** | **1** | | **2** | **3** |
| GS according to EULAR-OMERACT | |  |  | |  |  |
|  | **0** | 32 | 20 | | 0 | 0 |
|  | **1** | 0 | 8 | | 6 | 1 |
|  | **2** | 0 | 0 | | 0 | 0 |
|  | **3** | 0 | 0 | | 0 | 0 |
| **MTP 4** |  | GS according to Szkudlarek | | | | |
|  | | **0** | **1** | | **2** | **3** |
| GS according to EULAR-OMERACT | |  |  | |  |  |
|  | **0** | 45 | 11 | | 2 | 0 |
|  | **1** | 0 | 2 | | 5 | 0 |
|  | **2** | 0 | 0 | | 1 | 0 |
|  | **3** | 0 | 0 | | 0 | 0 |
| **MTP 5** |  | GS according to Szkudlarek | | | | |
|  | | **0** | **1** | | **2** | **3** |
| GS according to EULAR-OMERACT | |  |  | |  |  |
|  | **0** | 49 | 8 | | 3 | 0 |
|  | **1** | 0 | 2 | | 4 | 1 |
|  | **2** | 0 | 0 | | 0 | 0 |
|  | **3** | 0 | 0 | | 0 | 0 |

FIGURES

Figure S1. Grey-scale (according to EULAR-OMERACT definition; A,B,C) and power Doppler Ultrasound-detected synovitis (D,E,F) versus MRI-detected synovitis on MCP, wrist, and MTP joint level for IA.


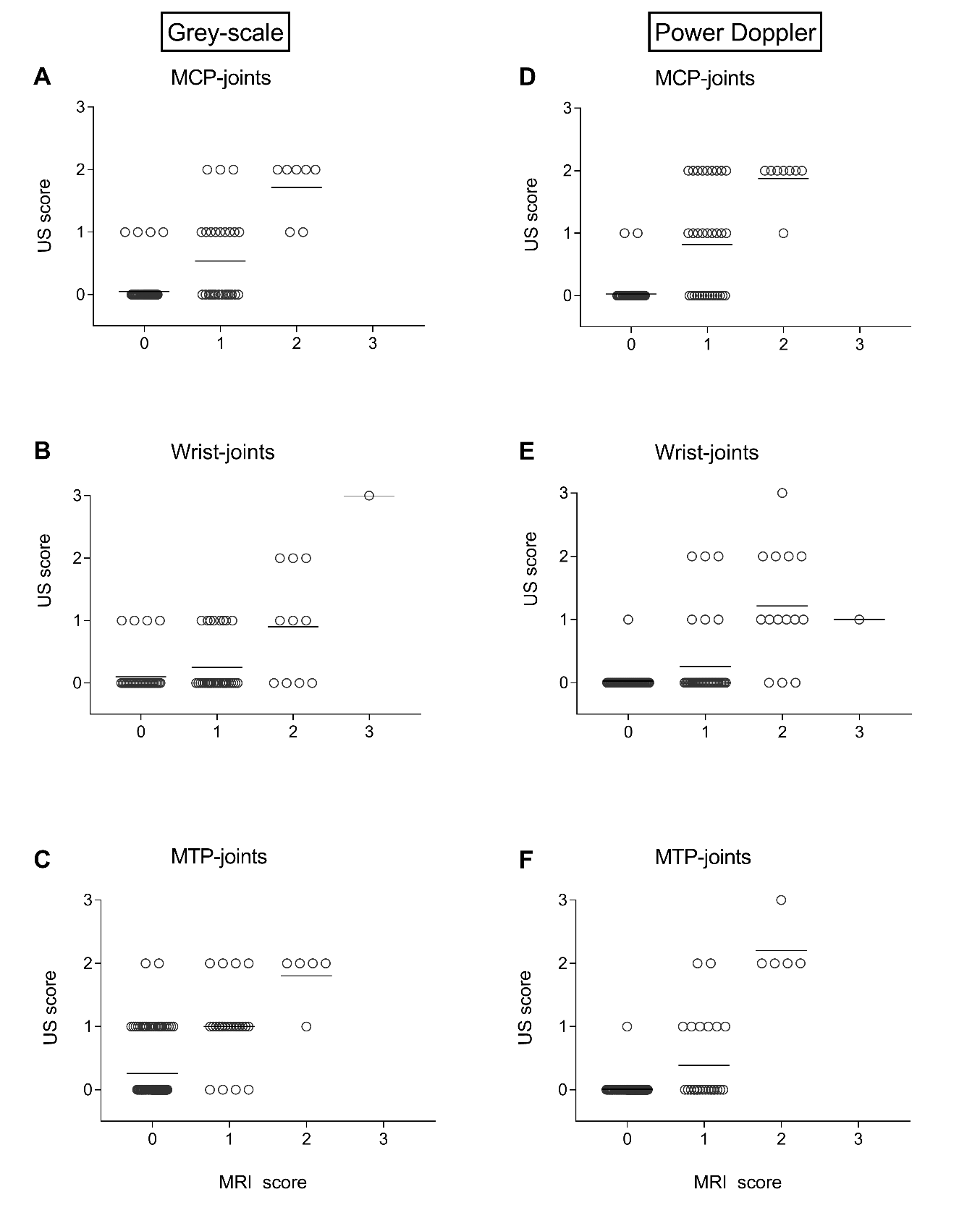


Figure S2. Grey-scale (according to EULAR-OMERACT definition; A,B,C) and power Doppler Ultrasound-detected synovitis (D,E,F) versus MRI-detected synovitis on MCP, wrist, and MTP joint level for CSA.


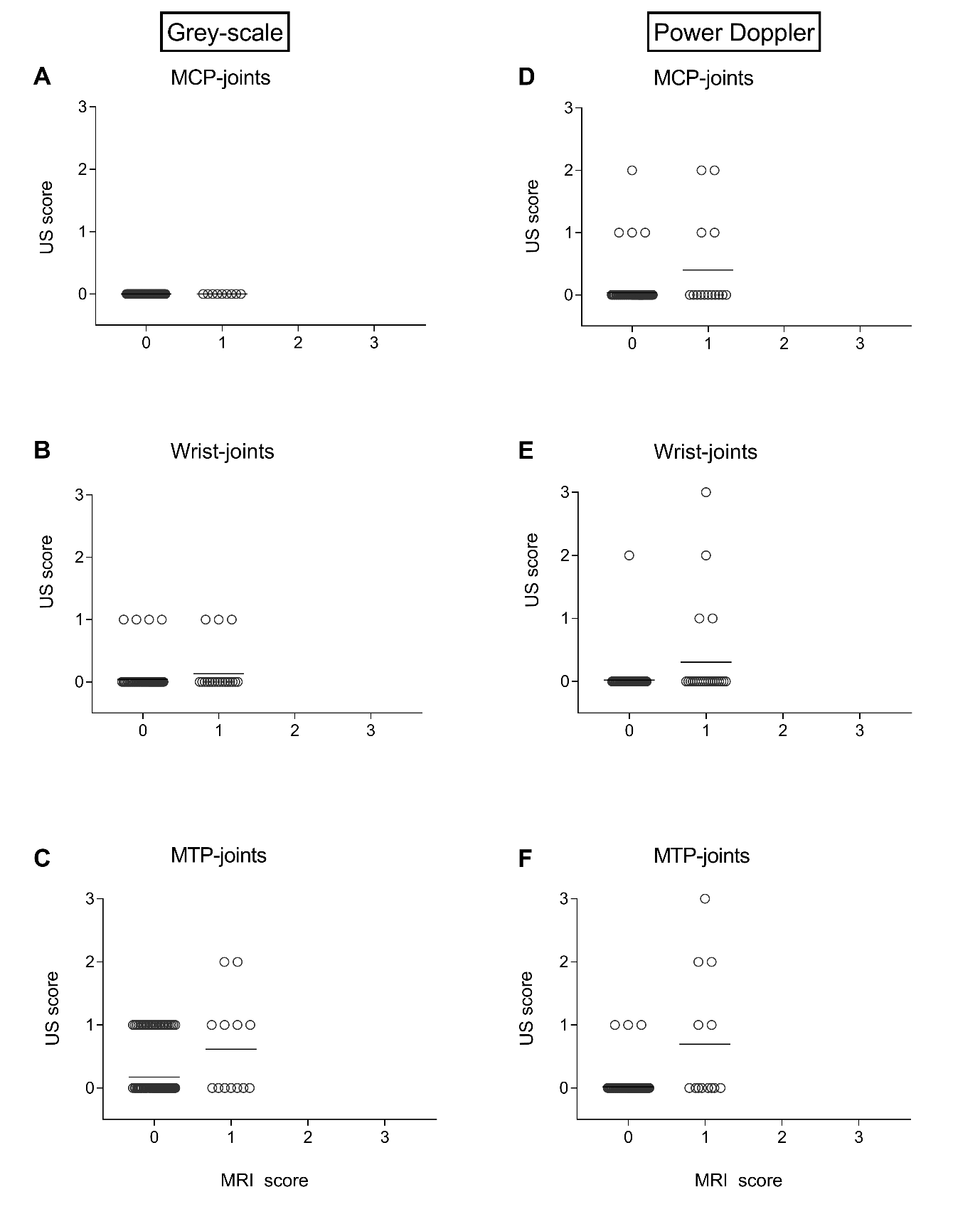


Figure S3. Grey-scale (A,B,C) and power Doppler Ultrasound-detected tenosynovitis (D,E,F) versus MRI-detected tenosynovitis of MCP flexor 2-5, wrist flexor and extensor tendons for IA.


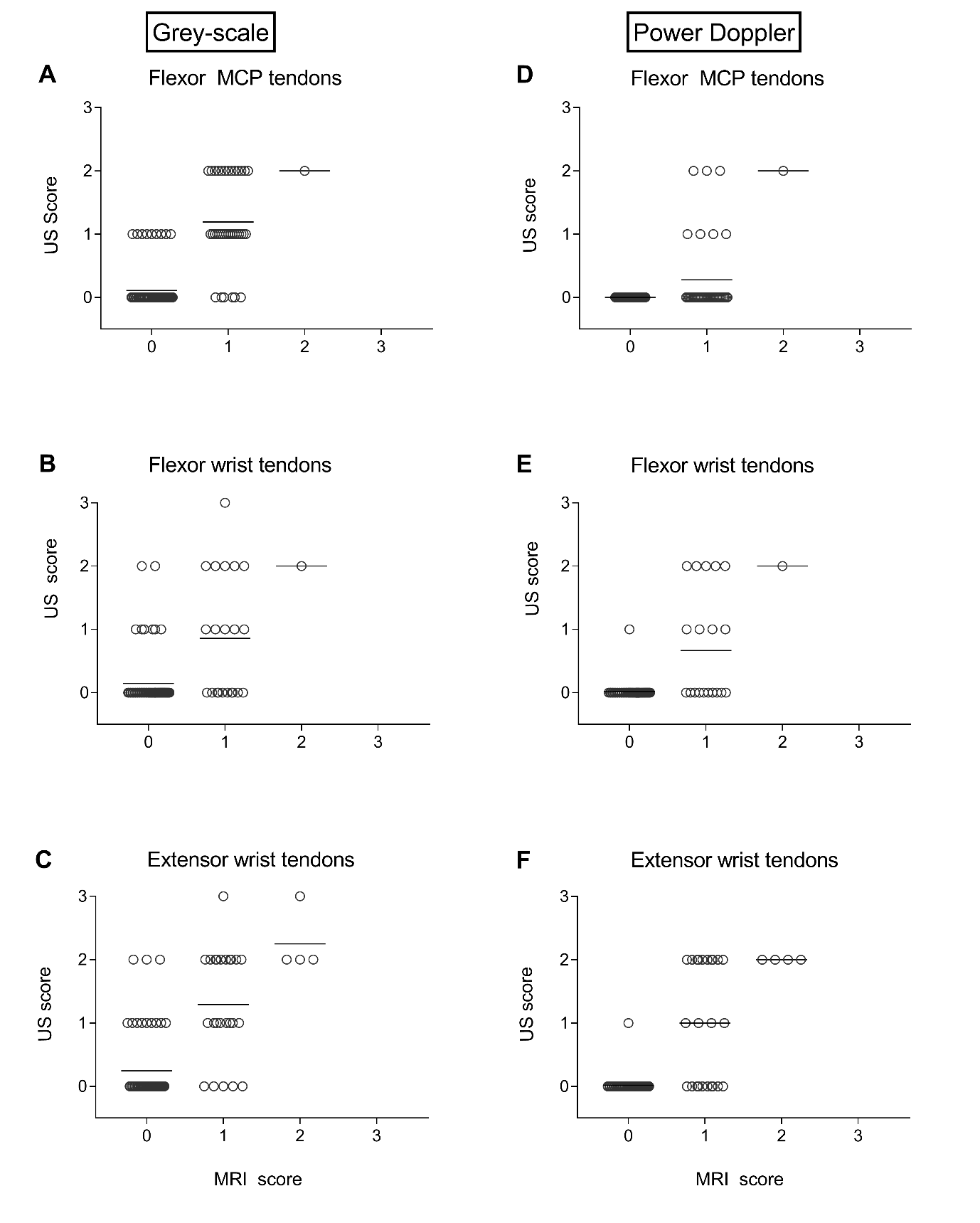


Figure S4. Grey-scale (A,B,C) and power Doppler Ultrasound-detected tenosynovitis (D,E,F) versus MRI-detected tenosynovitis of MCP flexor 2-5, wrist flexor and extensor tendons for CSA.


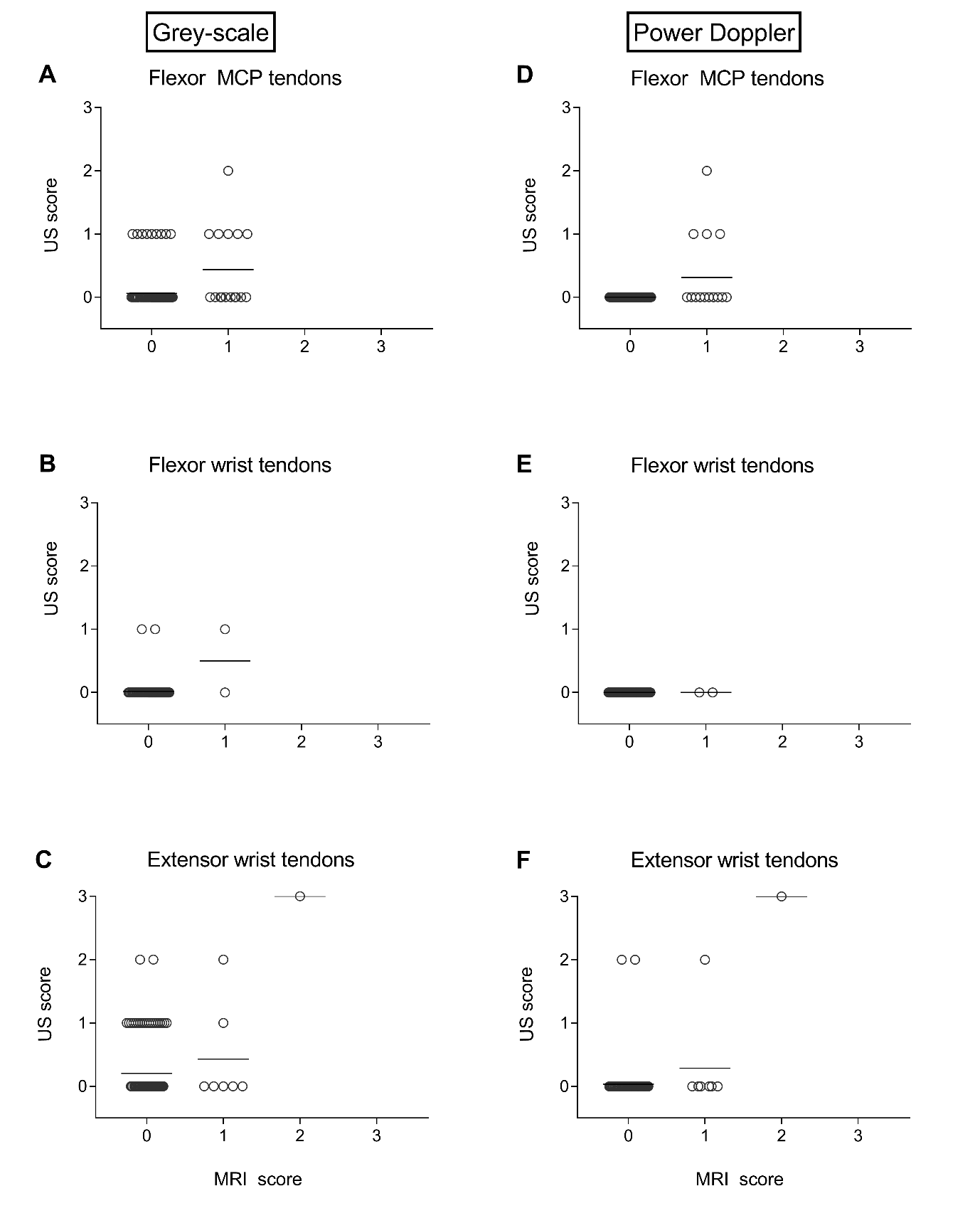


**SUPPLEMENTARY METHODS**

*Patients*

Inclusion in the EAC-cohort required having recent-onset clinically evident IA of ≥1 joint with a symptom duration <2 years, as described previously[4]. Inclusion in the CSA-cohort, which has the aim to study the symptomatic phase of RA preceding clinical arthritis, required the presence of arthralgia of small joints for <1 year which was, due to the character of the symptoms, considered as being suspect to progress to RA by a rheumatologist. CSA was identified at the first visit, before the results of routine laboratory investigations were known. A detailed description is provided elsewhere[5].

*Study protocol*

At inclusion, all patients had physical examination including 66-swollen and 68-tender joint counts. Laboratory investigations were performed and questionnaires completed. All patients underwent unilateral contrast enhanced MRI of metacarpophalangeal (MCP)-, wrist and metatarsophalangeal (MTP)-joints and musculoskeletal US at the same day <2weeks after first presentation.

*MRI protocol*

MR imaging was performed on a MSK-extreme 1.5T extremity MR imaging system (GE, Wisconsin, USA) using a 145mm coil for the foot and a 100mm coil for the hand. The patient was positioned in a chair beside the scanner, with the hand or foot fixed in the coil with cushions.

For all patients an MRI was made at baseline, with gadolinium contrast enhancement for metacarpophalangeal (MCP)-, wrist-and metatarsophalangeal (MTP)-joints. In the hand (MCP 2-5 and wrist) the following sequence was acquired before contrast administration: T1-weighted fast spin-echo (FSE) sequence in the coronal plane (repetition time (TR) 575ms, echo time (TE) 11.2ms, acquisition matrix 388×288, echo train length (ETL) 2). After intravenous injection of gadolinium contrast (gadoteric acid, Guerbet, Paris, France, standard dose of 0.1mmol/kg) the following sequences were obtained: T1-weighted FSE sequence with frequency selective fat saturation (fatsat) in the coronal plane (TR/TE 700/9.7ms, acquisition matrix 364×224, ETL 2), T1-weighted FSE sequence with frequency selective fat saturation in the axial plane (wrist: TR/TE 540/7.7ms; acquisition matrix 320x192; ETL 2 and MCP-joints: TR/TE 570/7.7ms; acquisition matrix 320x192; ETL 2). The obtained sequences of the forefoot (MTP 1-5 joints) were post-contrast imaging of the foot: T1-weighted FSE fatsat sequence in the axial plane (TR/TE 700/9.5ms; acquisition matrix 364x224, ETL 2) and: T1-weighted FSE fatsat sequence in the coronal plane (perpendicular to the axis of the MTP-joints) (TR/TE 540/7.5ms; acquisition matrix 320x192, ETL 2).

Field-of-view was 100mm for the hand and 140mm for the foot. Coronal sequences of the hand had 18 slices with a slice thickness of 2mm and a slice gap of 0.2mm. Coronal sequences of the foot had 20 slices with a slice thickness of 3mm and a slice gap of 0.3mm. All axial sequences had a slice thickness of 3mm and a slice gap of 0.3mm with 20 slices for the wrist, 16 for the MCP-joints and 14 for the foot.

*MRI scoring and dichotomizing*

All bones and joints were scored semi-quantitatively in line with the validated RA MRI scoring system (RAMRIS) and the protocol was identical for all patients as described in previous studies[4-6]. Synovitis was scored in line with the Outcome Measures in Rheumatology Clinical Trials (OMERACT) RAMRIS[7]. RAMRIS was not developed to score MTP-joints, however others have previously adapted the RAMRIS to score MTP-joints as well[8]. Tenosynovitis was scored according to the method described by Haavardsholm (also applied at the flexor and extensor tendons at the 2-5 MCP-joints; range 0-3). The synovitis score (range 0-3) was scored based on the volume of enhancing tissue in the synovial compartment (none, mild, moderate, severe) and the tenosynovitis-score (ranged 0-3) was based on the thickness of peritendinous effusion or synovial proliferation with contrast enhancement (normal, <2mm, 2-5mm, >5mm)[4,5].

Mean scores of two readers were calculated and rounded down to whole points and lesions were considered absent in case it was scored by only one reader. Thus, in case of a mean score of ≥1 MRI was considered positive for MRI-detected synovitis or tenosynovitis.

***MR readers***

Scoring was performed by independent and trained readers, blinded to clinical data. The between-reader intraclass correlation coefficients (ICC) of all scans were for MRI-detected synovitis scores 0.94, for tenosynovitis 0.97.

***Musculoskeletal ultrasound scanning and scoring***

A high-end US machine was used (GE Logiq E9, Genova, Italy) with a linear array transducer of 6-15MHz. PD was assessed with a pulse repetition frequency of 0.8kHz and gain was set to a level until background signal was removed. The presence of synovitis was assessed on a semi-quantitative scale (0-3) for GS/PD according to Szkudlarek *et al.*[1], and synovial effusion and hypertrophy were combined (called ‘modified Szkudlarek-method’)[2].

Tenosynovitis was examined on a semi-quantitative scale for GS/PD according to the OMERACT[9]. We examined the following tendons: extensor carpi radialis longus et brevis (ECR), extensor digitorum communis/indices proprius (EDC/IP), extensor carpi ulnaris (ECU), flexor carpi radialis (FCR), flexor pollicis longus (FPL), flexor digitorum superficialis and profundus (FDS/FDP), and flexor digitorum (FD) 2-5 on MCP-level. All US-scores per joint (synovitis) and tendon (tenosynovitis) ranged from 0-3.

The static images of US were re-scored for GS synovitis (according to the EULAR-OMERACT-scoring method) by two examiners (SO and RvdB, with an ICC 0.92).[3] The mean scores of two readers were calculated and rounded down to whole points and lesions were considered absent in case it was scored by only one reader. Thus, in case of a mean score of ≥1 US was considered positive for US-detected GS-synovitis.

***US readers***

Scoring was performed by independent and trained readers (SO and RvdB), blinded to clinical data. The between-reader intraclass correlation coefficients (ICC) of GS-synovitis (EULAR-OMERACT-method) was 0.92.

**REFERENCES**

1. Szkudlarek M, Court-Payen M, Jacobsen S, Klarlund M, Thomsen HS, Ostergaard M. Interobserver agreement in ultrasonography of the finger and toe joints in rheumatoid arthritis. Arthritis Rheum. 2003 Apr; 48(4):955-962.

2. Scheel AK, Hermann KG, Kahler E, Pasewaldt D, Fritz J, Hamm B, et al. A novel ultrasonographic synovitis scoring system suitable for analyzing finger joint inflammation in rheumatoid arthritis. Arthritis Rheum. 2005 Mar; 52(3):733-743.

3. D’Agostino M-A, Terslev L, Aegerter P, Backhaus M, Balint P, Bruyn GA, et al. Scoring ultrasound synovitis in rheumatoid arthritis: a EULAR-OMERACT ultrasound taskforce - Part 1: definition and development of a standardised, consensus-based scoring system. RMD Open. 2017; 3(1).

4. Nieuwenhuis WP, van Steenbergen HW, Mangnus L, Newsum EC, Bloem JL, Huizinga TWJ, et al. Evaluation of the diagnostic accuracy of hand and foot MRI for early Rheumatoid Arthritis. Rheumatology (Oxford). 2017 Aug 1; 56(8):1367-1377.

5. van Steenbergen HW, van Nies JA, Huizinga TW, Bloem JL, Reijnierse M, van der Helm-van Mil AH. Characterising arthralgia in the preclinical phase of rheumatoid arthritis using MRI. Ann Rheum Dis. 2015 Jun; 74(6):1225-1232.

6. Mangnus L, van Steenbergen HW, Reijnierse M, van der Helm-van Mil AH. Magnetic Resonance Imaging-Detected Features of Inflammation and Erosions in Symptom-Free Persons From the General Population. Arthritis Rheumatol. 2016 May 23.

7. Ostergaard M, Edmonds J, McQueen F, Peterfy C, Lassere M, Ejbjerg B, et al. An introduction to the EULAR-OMERACT rheumatoid arthritis MRI reference image atlas. Ann Rheum Dis. 2005 Feb; 64 Suppl 1:i3-7.

8. Haavardsholm EA, Ostergaard M, Ejbjerg BJ, Kvan NP, Kvien TK. Introduction of a novel magnetic resonance imaging tenosynovitis score for rheumatoid arthritis: reliability in a multireader longitudinal study. Ann Rheum Dis. 2007 Sep; 66(9):1216-1220.

9. Naredo E, D'Agostino MA, Wakefield RJ, Moller I, Balint PV, Filippucci E, et al. Reliability of a consensus-based ultrasound score for tenosynovitis in rheumatoid arthritis. Ann Rheum Dis. 2013 Aug; 72(8):1328-1334.
